# Critical dialogue: recent advancements toward omission of axillary lymph node dissection in clinically node positive breast cancer patients treated with neoadjuvant chemo-immuno therapy: impact on radiology practice

**DOI:** 10.1186/s13244-025-01912-y

**Published:** 2025-03-06

**Authors:** Florien J. G. van Amstel, Janine M. Simons, Vivianne C. G. Tjan-Heijnen, Marjolein L. Smidt, Thiemo J. A. van Nijnatten

**Affiliations:** 1 Department of Radiology and Nuclear Medicine, Maastricht University Medical Centre+, Maastricht, The Netherlands; 2 Department of Surgery, Maastricht University Medical Centre+, Maastricht, The Netherlands; 3 GROW – Research Institute for Oncology and Reproduction, Maastricht University, Maastricht, The Netherlands; 4 Department of Radiotherapy, Erasmus MC Cancer Institute, University Medical Center Rotterdam, Rotterdam, The Netherlands; 5 Department of Medical Oncology, Maastricht University Medical Center+, Maastricht, The Netherlands

**Keywords:** Breast cancer, Axillary lymph node staging, Axillary imaging

## Current practice and knowledge

Less invasive restaging procedures, compared to axillary lymph node dissection (ALND), have become the standard of care in clinically node positive (cN+) breast cancer patients treated with neoadjuvant systemic therapy (NST) in most institutions [1]. Due to the therapeutic effects of NST, initial cN+ patients can achieve an axillary pathologic complete response (pCR) and are thought not to benefit from an ALND [1]. Less invasive restaging aims to reduce surgical morbidity related to ALND but carries the risk of a false negative outcome (i.e., absence of cancer cells in resected axillary lymph nodes but presence of cancer cells in remaining axillary lymph nodes). The diagnostic accuracy of various less invasive restaging procedures has been studied and showed different false negative rates (FNR) [2]. Although these less invasive restaging procedures have been implemented in clinical practice, the oncologic safety of omitting ALND in terms of axillary recurrence has, so far, remained uncertain. Montagna et al studied this in cN+ patients who achieved an axillary pCR after NST diagnosed with sentinel lymph node biopsy (SLNB) with dual-tracer mapping or targeted axillary dissection (TAD) after which an ALND was omitted [3]. The multicenter retrospective cohort study included 1144 cN+ patients from 25 centers across 11 countries over an 8-year period. The study reported low axillary recurrence rates (ARR) at a median follow-up period of 3.5 years, with 3- and 5-year ARR of 0.65% (95% confidence interval (CI): 0.29–1.30%) and 1.0% (95% CI: 0.49–2.00%), respectively. Furthermore, at 3-year follow-up, there was no significant difference in ARR between patients treated with SLNB versus TAD (0.8% vs 0.5%, *p* = 0.55) [3].

## Advancements and new developments

Studies addressing the oncologic safety of omitting ALND in cN+ patients treated with NST with axillary pCR following less invasive restaging procedures are limited and have primarily been smaller retrospective single-center studies. These studies reported low ARR ranging from 0.6 to 3.2% [4–6]. However, limitations such as potential lack of diversity in patient populations, variation in institutional practices, or small sample sizes restrict the generalizability of their study findings. Current ongoing clinical trials like ATNEC (NCT04109079) investigate the oncologic safety of omission of ALND in cN+ patients treated with NST that achieve axillary pCR diagnosed with less invasive restaging procedures, with results being anticipated in 2030 [7]. Therefore, the results of Montagna et al should be appreciated in the context of contributing to the ongoing debate regarding the oncologic safety of omitting ALND in cN+ patients treated with NST with axillary pCR following less invasive restaging.

## Considerations for clinical practice

Following NST, the probability of achieving axillary pCR strongly depends on breast cancer subtype. Patients with hormone (HR)-/ human epidermal growth factor receptor 2 + (HER2) and triple negative (TN) subtype have a high chance of axillary pCR compared to patients with HR+/HER2− subtype: 60 and 48% versus 18% [8]. Since only patients with an axillary pCR were included in the study of Montagna et al, a skewed distribution of breast cancer subtypes is seen: 54% (619/1144) had HER2+ subtype, 23% (262/1144) had TN subtype and 23% (263/1144) had HR+/HER2− subtype [3]. This limits the external validity of their findings. A more robust approach would be to analyze the oncologic safety of omitting ALND separately for each subtype.

As Montagna et al also mention, efforts should be made to perform restaging procedures with high accuracy and low FNR. Previous studies showed an unacceptable FNR exceeding 10% for the SLNB procedure in cN+ patients treated with NST, but extending the SLNB with dual-tracer mapping and removal of ≥ 3 sentinel lymph nodes (SLNs) led to a FNR below 10% [2]. TAD is associated with the lowest FNR, ranging from 2 to 4% [2, 9, 10]. Montagna et al show that, despite its higher FNR, an SLNB with dual-tracer mapping and removal of ≥ 3 SLNs may be an acceptable alternative to TAD since there was no significant difference in ARR after 3-year follow-up (0.8% for SLNB vs 0.5% for TAD, *p* = 0.55) [3]. Important to mention is that Montagna et al’s inclusion criteria were selective, with some centers only selecting patients with a clinical complete response after NST for SLNB or TAD. Potentially causing a false optimistic result, with lower FNRs that may not be applicable to patients without a clinical response but with axillary pCR. Consequently, future prospective research with longer follow-up periods is necessary to confirm whether SLNB and TAD indeed show similar oncological outcomes despite differences in FNR.

Considering axillary radiotherapy (ART), little data is available regarding the oncologic safety of omitting ART in cN+ patients who achieve axillary pCR after NST. At the 2023 San Antonio Breast Cancer Conference, preliminary results of the NSABP B-51/RTOG (NCT01872975) randomized controlled trial were presented [11]. In this trial, omitting ART was not associated with inferior oncological outcomes at 5 years in cN1 patients treated with NST who achieved axillary pCR. Importantly, about 50% of the patients underwent ALND in this trial [11]. Although none of the included patients underwent ALND in the study by Montagna et al, about 80% were, in fact, treated with ART [3]. Nonetheless, ART may still cause morbidity [12], and efforts should be made to determine in which cN+ patients who achieve axillary pCR both ALND and ART can be safely omitted.

## Considerations for radiology practice

Montagna et al support omission of ALND in cN+ patients treated with NST who achieve axillary pCR assessed by SLNB or TAD [3]. However, since current imaging modalities remain insufficiently accurate to reliably confirm axillary pCR following NST, less invasive restaging procedures remain the standard for all cN+ patients to accurately assess nodal status [13]. The high number of false positives may be reduced by refining radiologic assessments prior to surgery. One way to achieve this is by increasing the understanding that different breast cancer subtypes present different imaging characteristics [14]. Improved awareness of breast cancer subtypes may enhance the accuracy of radiologic response assessments, which is particularly important considering the significant variability in response rates to NST [8].
